# Salvage or Second Autologous SCT in Relapsed Multiple Myeloma (2016–2026): A Decade in Review

**DOI:** 10.3390/curroncol33030140

**Published:** 2026-02-28

**Authors:** Marwa Nassar, Nourah Alzaidy, Abdulrahman Nasiri, Amr Hanbali, Mahmoud A. Aljurf, Mostafa F. Mohammed Saleh

**Affiliations:** 1 Adult Hematology, Transplantation and Cellular Therapy Department, Oncology Center, King Faisal Specialist Hospital and Research Center, Riyadh 12713, Saudi Arabia; 2 College of Medicine, Imam Mohammad Ibn Saud Islamic University (IMSIU), Riyadh 11432, Saudi Arabia; 3 Clinical Hematology Unit, Department of Internal Medicine, Faculty of Medicine, Assiut University, Assiut 71515, Egypt

**Keywords:** multiple myeloma, salvage autologous stem cell transplantation, relapsed disease, maintenance therapy

## Abstract

**Background:** The role of second or salvage autologous stem cell transplantation (ASCT2) in relapsed multiple myeloma (MM) has evolved substantially over the past decade with the introduction of novel agents, maintenance strategies, and cellular immunotherapies. The clinical value of ASCT2 in the contemporary era requires reappraisal based on modern real-world and prospective data. **Methods:** We conducted a targeted literature review of PubMed-indexed studies published between January 2016 and January 2026 evaluating second or salvage autologous transplantation in adult patients with relapsed or refractory multiple myeloma. Retrospective registry analyses, real-world cohorts, and prospective randomized trials were included, focusing on feasibility, toxicity, progression-free survival (PFS), overall survival (OS), and prognostic determinants. Fourteen key studies formed the core evidence base, supplemented by guideline statements and comparative immunotherapy data. **Results:** Across large registry and institutional series, ASCT2 was consistently feasible with low non-relapse mortality (≤5% at day 100–1 year). The median PFS ranged from 9.8 to 30.2 months and the median OS from approximately 30 to >80 months, with outcomes strongly influenced by duration of remission after first ASCT. Patients relapsing ≥24 months after ASCT1 derived the greatest benefit, achieving a median PFS of 17–45 months and OS exceeding 60 months in favorable-risk subgroups. Post-ASCT2 maintenance, particularly with lenalidomide or carfilzomib-based regimens, significantly prolonged disease control in randomized and real-world studies. Conversely, the phase III GMMG ReLApsE trial did not demonstrate a survival advantage for routine salvage ASCT over continuous lenalidomide-based therapy, highlighting the importance of patient selection. In heavily refractory or cytopenic populations, ASCT2 provided modest disease control but enabled hematopoietic recovery and access to subsequent therapies. **Conclusions:** In the modern treatment landscape, second or salvage autologous transplantation remains a valid, safe, and effective strategy for carefully selected patients with relapsed multiple myeloma, particularly those with chemosensitive disease and prolonged initial remissions. ASCT2 should be integrated in a risk-adapted manner alongside maintenance therapy and emerging immunotherapies, serving as a durable consolidation or bridging approach rather than routine therapy for all relapsed patients.

## Simple Summary

Multiple myeloma is a blood cancer that often returns after initial treatment, which can include a stem cell transplant. For these relapsed patients, a second transplant is a possible treatment, but its value is being re-evaluated as newer immune-based therapies become available. This review examines research from the past decade and confirms that a second transplant remains a safe and effective choice for carefully selected patients. The best results are seen in patients who had a long period of remission after their first transplant. Our findings suggest that a second transplant should be considered a personalized tool, used alongside other modern treatments, rather than a routine procedure for every relapsed patient. This highlights the need for studies directly comparing it to new immunotherapies.

## 1. Introduction

High-dose melphalan followed by autologous stem cell transplantation (ASCT) remains a core component of frontline therapy for transplant-eligible multiple myeloma (MM), yet most patients ultimately relapse and require subsequent lines of therapy [1]. With the rapid expansion of proteasome inhibitors, IMiDs, anti-CD38 monoclonal antibodies, and more recently CAR T-cell and bispecific antibody platforms, the place of “classical” salvage strategies—especially second (ASCT2) or delayed/salvage autologous hematopoietic cell transplantation (auto-HCT)—has been increasingly questioned [2].

Despite this, ASCT2 retains several practical advantages: it is a fixed-duration intervention, widely available, and can deliver meaningful cytoreduction in chemosensitive relapse, potentially serving as consolidation after effective re-induction or as a “bridge” when access to cellular therapies is delayed. Contemporary series also suggest that treatment-related mortality remains low (generally ≤1–4% at day 100), even in heavily pretreated patients, supporting feasibility in selected populations [3].

The key clinical challenge is selection: multiple datasets consistently identify remission duration after ASCT1 (commonly ≥18–24 months) as the dominant predictor of benefit from ASCT2, while high-risk cytogenetics, advanced stage, refractoriness to key drug classes, and heavy prior therapy reduce durability. In parallel, randomized evidence versus modern non-transplant strategies is limited and mixed, reinforcing the need for risk-adapted integration rather than routine application.

## 2. Methods

A targeted literature search was conducted using PubMed to identify studies published between 1 January 2016, and 29 January 2026, evaluating the role of second or salvage autologous hematopoietic stem cell transplantation in relapsed multiple myeloma. Search terms were used in various combinations and included multiple myeloma, second autologous transplant, salvage autologous, retransplant, delayed autologous transplant, maintenance after salvage ASCT, and high-dose melphalan. The literature search was conducted independently by two authors (M.N. and N.A.) to minimize selection bias, with discrepancies resolved by consensus. The search strategy was designed to capture studies performed in the modern treatment era and prioritized reports providing clinically relevant outcomes, including progression-free survival (PFS) or time to progression (TTP), overall survival (OS), non-relapse or treatment-related mortality (NRM/TRM), and depth of response.

Studies were eligible for inclusion if they met the following criteria: (i) enrolled adult patients with relapsed or refractory multiple myeloma undergoing a second autologous transplantation, either as salvage or delayed consolidation; (ii) evaluated prognostic or predictive factors, such as interval from first ASCT, disease stage, cytogenetic risk, or refractoriness to prior therapies; and/or (iii) reported outcomes of post-ASCT2 maintenance strategies. Retrospective single-center studies, multicenter registry analyses, and prospective randomized trials were included when available. Pediatric-only studies, case reports, and publications lacking outcome data were excluded. To ensure consistency with the predefined reference framework, all studies listed in the reference set were incorporated, with the Results section focused on the first 13 studies.

Given the heterogeneity across study designs, patient populations, treatment eras, and comparator strategies—including differences between single-center and registry-based analyses, salvage versus delayed transplantation, and variability in induction and maintenance regimens—a narrative synthesis approach was adopted. It is important to acknowledge the potential for selection bias inherent in this narrative review methodology. Emphasis was placed on three reproducible domains across studies: (1) feasibility and toxicity of salvage or second ASCT; (2) efficacy benchmarks defined by median PFS and OS; and (3) consistent selection factors associated with benefit, particularly the duration of remission following the first ASCT.

## 3. Results

Across studies published over the last decade, ASCT2 in relapsed multiple myeloma was consistently shown to be feasible, with low treatment-related mortality and clinically meaningful disease control in selected patients (Table 1). The available evidence comprised retrospective single-center experiences, large registry-based analyses, and limited prospective randomized data, allowing assessment of safety, efficacy benchmarks, prognostic determinants, and the impact of post-transplant maintenance. Collectively, these studies delineate patient subsets most likely to benefit from ASCT2, while also defining scenarios in which its advantage over non-transplant strategies is limited.

Gössi et al. analyzed 86 consecutive patients with multiple myeloma at first relapse after ASCT1 and demonstrated a clinically meaningful advantage for ASCT2 in appropriately selected patients. In this cohort, 61 patients (71%) underwent ASCT2 and achieved significantly improved outcomes compared with conventional chemotherapy, including a longer median PFS (30.2 vs. 13.0 months; *p* = 0.026) and markedly prolonged OS (129.6 vs. 33.5 months; *p* = 0.0003). Benefit was greatest in patients relapsing >12 months after ASCT1, with improved post-relapse PFS2 (*p* = 0.0179) and OS2 (*p* = 0.0009). Lenalidomide maintenance after ASCT2 further enhanced outcomes, extending the median PFS to 41.0 months compared with 21.6 months without maintenance (*p* = 0.0034), with improved OS (not reached vs. 129.6 months; *p* = 0.0434) [4].

Prospective randomized evidence supporting salvage ASCT was provided by the phase III Myeloma X Relapse (Intensive) trial. In this study, Cook et al. randomized 174 patients with relapsed multiple myeloma after prior ASCT to receive either high-dose melphalan with salvage ASCT or weekly oral cyclophosphamide following bortezomib-based re-induction. Salvage ASCT resulted in a significantly longer median time to disease progression (19 vs. 11 months; HR = 0.45, *p* < 0.0001) and translated into a durable overall survival advantage, with a median OS of 67 months compared with 52 months in the chemotherapy arm (HR = 0.56, *p* = 0.0169). Time to second progression was also markedly prolonged (67 vs. 35 months; HR = 0.37, *p* < 0.0001). Treatment-related mortality remained low, and the cumulative incidence of second primary malignancies at 60 months was 5.2%, supporting the safety and long-term benefit of salvage ASCT when applied at first relapse [5].

Large registry and institutional series confirmed feasibility and safety of salvage or delayed auto-HCT, with consistently low non-relapse mortality and durable outcomes in patients with longer first remissions. In the EBMT analysis by Drozd-Sokołowska et al. (n = 305), salvage auto-HCT performed using remobilized stem cells after prior auto-HCT was feasible, with low NRM and relapse as the predominant cause of failure [2]. In the CIBMTR report by Lemieux et al. (n = 975), NRM remained low (2% at day 100; 4% at 1 year), with a median PFS of 9.8 months and a median OS of 30.9 months; outcomes were strongly dependent on the ASCT1-to-relapse interval, with patients relapsing ≥24 months achieving superior PFS (17.3 months) and OS (71.3 months) compared with those relapsing earlier (PFS 9.8 months; OS 28.5 months) [6]. In the largest single-center cohort, Pasvolsky et al. evaluated 650 patients undergoing delayed (n = 335) or salvage (n = 315) autoHCT and reported low NRM (3% at day 100; 4% at 1 year), with a median PFS of 17.5 months and OS of 47.3 months, and no significant difference between delayed and salvage approaches; within the salvage subgroup, transplant performed ≥24 months after ASCT1 was associated with superior outcomes [3].

Contemporary real-world series further refined prognostic stratification and highlighted the importance of clinical risk, chemosensitivity, and depth of response. Karp et al. reported outcomes of re-AHCT in 171 cases with a median PFS of 20.6 months, a median OS of 65.0 months, and 100-day mortality of 4%; R-ISS stage II/III and duration of response ≤24 months after the previous autologous transplant were independent adverse prognostic factors, whereas a favorable subgroup (R-ISS I and DoR >24 months) achieved prolonged PFS (45.0 months) and OS (80.2 months) [7]. In the Hungarian single-center study by Bicskó et al. (n = 30), TRM was 3%, median PFS 24 months, and median OS 48 months, with outcomes strongly associated with day-100 response, as patients achieving CR or VGPR had a median PFS of 32 months versus 8.5 months in those remaining in PR (*p* = 0.0006) [10]. Similarly, in the French multicenter real-life analysis by André et al. (n = 267), early mortality was 1%, ORR after ASCT2 was 96%, and 78% achieved ≥VGPR; the median EFS was 2.6 years and the median OS reached 8.1 years, with achievement of VGPR+ after ASCT2 and receipt of maintenance therapy emerging as the strongest predictors of improved OS (time-dependent HR 0.4 and 0.2, respectively) [11].

Specific subgroups and prospective data clarified the boundaries of benefit and underscored the importance of post-transplant strategies. Sever et al. evaluated 877 patients undergoing auto-HCT2 in the EBMT CALM registry, including 61 poor mobilizers who were successfully remobilized using plerixafor-based (39%) or non-plerixafor approaches (61%); engraftment kinetics, PFS, and OS were comparable between poor and non-poor mobilizers, with a median OS of approximately 41 months in both groups, supporting the feasibility of auto-HCT2 even in patients with inadequate initial mobilization [8]. In the Slovenian cohort by Čemažar et al. (n = 78), outcomes were favorable, with a median PFS of 24 months (95% CI 20–36), median OS of 76 months (95% CI 48–NR), day-100 ORR of 85%, and ≥VGPR rate of 65% [2]. In contrast, the long-term follow-up of the phase III GMMG ReLApsE trial (Baertsch et al.; n = 277) demonstrated no significant PFS or OS advantage for a strategy incorporating salvage ASCT plus lenalidomide maintenance compared with continuous LEN/DEX (median PFS 20.5 vs. 19.3 months; HR = 0.98; *p* = 0.90; median OS 67.1 vs. 62.7 months; HR = 0.89; *p* = 0.44), although non-receipt of ASCT and crossover to transplant likely attenuated the observed effect [14]. Evidence supporting maintenance after salvage ASCT was strengthened by the Nordic randomized phase II CARFI trial (Gregersen et al.; 168 randomized), in which carfilzomib–dexamethasone maintenance significantly prolonged time to progression compared with observation (median 25.1 vs. 16.7 months; HR = 0.46; *p* = 0.0004), with low day-100 mortality (~1%) and manageable toxicity [15].

## 4. Discussion

This review synthesizes evidence published over the last decade to critically appraise the role of second or salvage autologous stem cell transplantation in relapsed multiple myeloma. By systematically integrating outcomes from retrospective cohorts, registry analyses, and prospective trials, it aims to clarify where salvage ASCT remains clinically meaningful in contemporary practice. The findings are interpreted in the context of rapidly expanding immunotherapeutic options and evolving treatment sequencing.

Feasibility and safety remain consistent across modern series. Contemporary cohorts and registry datasets repeatedly demonstrate low early mortality for ASCT2/salvage auto-HCT, typically ~1–4% at day 100 or early post transplant, even in heavily pretreated populations [3,7,10,11]. For example, Pasvolsky et al. reported non-relapse mortality (NRM) of 3% at day 100 and 4% at 1 year in 650 delayed/salvage autoHCTs [3], and Lemieux et al. reported NRM of 2% at day 100 and 4% at 1 year in 975 CIBMTR cases [9]. These rates compare favorably with the higher NRM historically observed with allogeneic transplant approaches, even with reduced-intensity conditioning strategies [16].

Relapse remains the dominant driver of failure, underscoring the central importance of patient selection. Registry studies consistently show that relapse, rather than toxicity, accounts for the majority of adverse outcomes. In the EBMT analysis by Drozd-Sokołowska et al., relapse incidence reached 56% at 2 years and 76% at 4 years after salvage auto-HCT performed using remobilized stem cells [6]. This pattern supports guidance from international consensus statements that salvage ASCT should be offered to patients with chemosensitive disease and the highest likelihood of durable disease control, rather than reserved for late, multi-refractory settings [17].

The ASCT1-to-relapse interval is the most reproducible predictor of benefit. Across independent cohorts, prolonged remission after ASCT1 is consistently associated with superior outcomes after ASCT2, serving as a pragmatic surrogate for disease biology and therapy responsiveness. Gössi et al. found that patients relapsing >12 months after ASCT1 derived the greatest benefit from ASCT2, with significantly improved PFS2 and OS2 [4]. Similarly, Lemieux et al. reported that patients relapsing ≥24 months after ASCT1 achieved a median PFS of 17.3 months and OS of 71.3 months, compared with 9.8 months and 28.5 months in those relapsing earlier [9]. Pasvolsky et al. also found that a transplant performed ≥24 months after ASCT1 was associated with superior outcomes [3]. This distinction highlights that while ASCT2 can be effective in later lines of therapy, its competitiveness is significantly greater in earlier relapse, particularly for patients who have demonstrated a durable response to initial therapy.

Further refinement of patient selection can be achieved by incorporating prognostic markers such as MRD, cytogenetic risk, and refractoriness to specific drug classes. While not uniformly reported across all studies, emerging data clarify the impact of these factors. For patients undergoing a second-line ASCT, achieving MRD negativity is a powerful predictor of outcome, associated with a significantly better 3-year PFS compared to those who remain MRD-positive (71% vs. 27%) [18]. However, MRD negativity may not fully overcome the adverse prognosis of high-risk cytogenetics. Even in MRD-negative patients, the presence of del(17p), t(4;14), or 1q21+ is associated with a shorter PFS, underscoring the persistent impact of underlying disease biology [19]. Furthermore, refractoriness to key drug classes is a major negative prognostic factor. In patients with daratumumab-refractory disease, salvage ASCT can induce responses but with limited durability, suggesting its role is primarily as a bridge to other therapies in this challenging population [12].

Post-ASCT2 maintenance therapy significantly improves outcomes. Both lenalidomide and carfilzomib-based maintenance have been shown to prolong disease control. Gössi et al. demonstrated that lenalidomide maintenance extended median PFS to 41.0 months from 21.6 months [4]. The Nordic CARFI trial showed that carfilzomib–dexamethasone maintenance significantly prolonged time to progression (25.1 vs. 16.7 months) [15]. It is noteworthy that in the CARFI trial, only one-third of the patients were lenalidomide-exposed, a scenario that is becoming increasingly less frequent in current practice.

Randomized comparisons have not uniformly demonstrated superiority of salvage-ASCT strategies, and interpretation requires attention to treatment delivery and crossover. In the long-term follow-up of the phase III GMMG ReLApsE trial, a LEN/DEX → ASCT → lenalidomide maintenance strategy did not improve PFS (20.5 vs. 19.3 months; HR = 0.98; *p* = 0.90) or OS (67.1 vs. 62.7 months; HR = 0.89; *p* = 0.44) compared with continuous LEN/DEX [14]. Notably, 29% of patients assigned to transplant did not proceed to ASCT and 35% of controls crossed over to ASCT later, factors that plausibly attenuate measurable differences. These realities mirror routine practice, where clinical deterioration, logistics, and evolving salvage options frequently determine whether ASCT2 is delivered, emphasizing the distinction between evaluating “strategy” versus “procedure”.

Mobilization feasibility supports salvage ASCT2 even in challenging settings, while health system data suggest that stem cell collection policies warrant re-evaluation. In the EBMT CALM registry analysis, Sever et al. showed that poor mobilization did not preclude auto-HCT2; with plerixafor-based remobilization used in 39%, engraftment and survival were comparable to non-poor mobilizers and the median OS was approximately 41 months in both groups [8]. Conversely, cost/utilization studies indicate that routine collection and long-term storage of excess cells for future salvage is often inefficient. Chhabra et al. reported that only 12% of patients underwent salvage autoHCT by 6 years, with an added mean cost of approximately $10,800 per patient for extra collection and only ~14% utilization for salvage [20]. Yassine et al. similarly reported very low utilization of stored cells for salvage auto-HCT (1.4%), with high storage-associated costs (median $23,840 per patient) and approximately 70% of stored cells never used [21]. Together, these findings support a more individualized, risk-adapted approach to collection targets rather than universal harvesting for multiple future transplants. 

Beyond late-relapse chemosensitive disease, ASCT2 may serve enabling or bridging functions in selected refractory or cytopenic patients. Although late relapsers derive the clearest durable benefit, some high-risk populations may benefit from cytoreduction and hematopoietic recovery. Tremblay et al. reported that despite extensive pretreatment and baseline cytopenias, 72% of thrombocytopenic and 64% of neutropenic patients recovered counts after ASCT2, enabling 37% to subsequently enroll in clinical trials; NRM was 4% at day 100, although the median PFS and OS were modest (6.1 and 19.3 months) [13]. In daratumumab-refractory disease, Yarlagadda et al. observed an ORR of 80% with CR 43% and MRD negativity 45% (10^−5^), but the median PFS and OS remained limited (7.3 and 19.3 months), supporting salvage ASCT as a bridge or disease-stabilizing option rather than a definitive approach in highly refractory settings [12].

Benchmarking salvage ASCT against modern immunotherapies clarifies its niche in contemporary algorithms. Anti-CD38 antibody-based combinations deliver an ORR of 60–90% and a median PFS commonly of 12 to >30 months depending on the line of therapy and regimen [22]. CAR-T therapies achieve high response rates but introduce manufacturing delays and distinctive toxicity; ide-cel in KarMMa achieved an ORR of 73% (CR 33%) with a median PFS of 8.8 months and OS of 19.4 months, whereas cilta-cel in CARTITUDE-1 produced an ORR of ~97–98% with ≥CR of ~80–83% and ~2-year PFS of ~60% [23]. In this context, ASCT2 competes less on maximal response rates and more on accessibility, fixed duration, and predictable early safety, particularly for patients with late relapse and preserved chemosensitivity.

Bispecific antibodies and combinations raise efficacy expectations but increase infectious morbidity, making sequencing and supportive care central. Teclistamab in MajesTEC-1 produced an ORR of ~63% and ≥CR of ~39% with a median PFS of ~11.3 months, with infections frequent and often severe (grade ≥3 approximately ~45% in many series) [24]. Targeting an alternative antigen, talquetamab (GPRC5D×CD3) in the MonumenTAL-1 study demonstrated similarly high activity in heavily pretreated disease, with ORRs of 69–74% across recommended subcutaneous dosing cohorts, including responses in patients previously exposed to BCMA-directed therapies; median duration of response ranged from 7.8 to 10.2 months, but infections occurred in 59–76% of patients and grade 3–4 cytopenias were common [25,26]. In earlier relapse, teclistamab–daratumumab in MajesTEC-3 achieved an estimated 36-month PFS of 83.4% versus 29.7% with DPd/DVd (HR = 0.17) and MRD negativity (10^−5^) of 58.4% versus 17.1% [27]. Dual-bispecific targeting with talquetamab plus teclistamab achieved an ORR of 80% (including 61% in extramedullary disease), but grade 3–4 infections reached 64% [28]. These data indicate that while immunotherapy-based strategies can provide unprecedented disease control, they also necessitate robust infection mitigation and may not be universally deliverable, preserving a role for salvage ASCT in selected settings.

**Figure 1:**
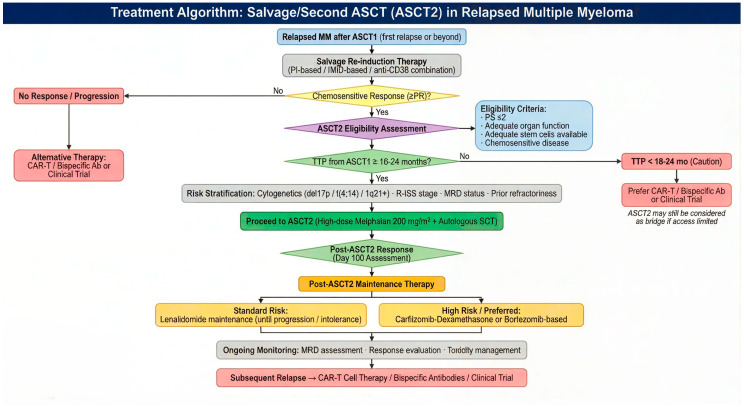
Proposed risk-adapted treatment algorithm for salvage or second autologous stem cell transplantation (ASCT2) in relapsed multiple myeloma.

Based on the totality of evidence reviewed, we propose a risk-adapted treatment algorithm for the integration of ASCT2 in the management of relapsed multiple myeloma (Figure 1).

Real-world bridging strategies increasingly rely on bispecific antibodies, yet ASCT2 remains relevant where access is limited or hematologic rescue is needed. Dhakal et al. reported that talquetamab bridging achieved a 71% response rate and enabled 89% of patients to proceed to BCMA CAR-T, with no grade ≥3 CRS and 2% grade 3 ICANS; post-CAR-T ORR remained 88% with a CR of 54% [29]. These findings support pharmacologic bridging to CAR-T in contemporary practice, although ASCT2 may still serve as a fixed-duration bridge in centers without ready access to bispecifics or when marrow reserve and cytopenias are limiting, as suggested by the hematopoietic recovery observed in cytopenic cohorts [13]. Practical recommendations from the European Myeloma Network favor CAR-T first when patients are eligible for both modalities, given concerns that prior bispecific exposure may reduce subsequent CAR-T efficacy [30].

Overall, contemporary evidence supports ASCT2/salvage auto-HCT as a risk-adapted option for selected patients in the modern era. The most consistent signals favoring benefit include a prolonged remission after ASCT1 (generally ≥18–24 months), chemosensitive relapse with meaningful response to re-induction, and achievement of deep response following ASCT2. In parallel, the expanding efficacy of CAR-T cells and bispecific antibodies along with sequencing guidance that prioritizes CAR-T when feasible will continue to narrow ASCT2 toward clearly defined niches: consolidation for late relapse, resource-efficient fixed-duration therapy when cellular immunotherapy access is constrained, and a benchmark comparator in real-world effectiveness and cost–utility analyses.

## 5. Conclusions

ASCT2 remains a safe and effective option for selected patients with RRMM in the contemporary treatment era. Clinical benefit is most pronounced in patients with chemosensitive relapse and a prolonged interval after ASCT1, particularly when ASCT2 is followed by maintenance therapy. In the context of expanding immunotherapeutic strategies, ASCT2 should be applied in a risk-adapted manner as consolidation or bridging therapy rather than routine salvage for all relapsed patients.

However, there is a notable lack of research on salvage ASCT combined with newer drug combinations such as re-induction therapy. Furthermore, the heterogeneity of patient characteristics across the available studies makes direct comparisons challenging. There are few prospective studies, and, most importantly, no prospective randomized trials comparing salvage ASCT with modern immunotherapies, which represents a critical knowledge gap.

## Figures and Tables

**Table 1:** Key studies evaluating second or salvage autologous stem cell transplantation (ASCT2) in relapsed multiple myeloma.

Study (Year)/ Data Source	Study Design	N (ASCT 2)	Clinical Setting	Median PFS (Months)	Median OS (Months)	NRM/TRM	Key Findings
Gössi et al., 2018 [4]	Single-center retrospective	61 (of 86)	First relapse after ASCT1	30.2 vs. 13.0 (CT)	129.6 vs. 33.5	NR	ASCT2 significantly improved PFS and OS; lenalidomide maintenance prolonged PFS to 41.0 months
Cook et al., 2016 (Myeloma X Relapse) [5]	Phase III randomized	89 (of 174)	First relapse after ASCT1	TTP 19 vs. 11 (CT)	67 vs. 52	NR	Salvage ASCT at first relapse improved disease control and OS versus chemotherapy consolidation
Drozd-Sokołowska et al., 2022 (EBMT) [6]	Multicenter registry	305	Salvage ASCT with remobilized cells	15% at 4 y	52% at 4 y	5% (2 y); 9% (4 y)	Salvage ASCT feasible with remobilized cells; longer interval from ASCT1 predicted better OS/PFS
Karp et al., 2025 [7]	Two-center retrospective	171	RRMM, novel-agent era	20.6	65.0	4% (100 d)	R-ISS II/III and DoR ≤24 mo adverse; favorable subgroup achieved PFS 45 mo and OS 80 mo
Pasvolsky et al., 2025 [3]	Single-center retrospective	650	Delayed vs. salvage ASCT	17.5	47.3	3% (100 d); 4% (1 y)	No difference between delayed and salvage ASCT; ≥24 mo interval from ASCT1 strongly prognostic
Sever et al., 2025 (EBMT CALM) [8]	Prospective registry analysis	877 (61 PM)	ASCT2 in poor mobilizers	9.6 (PM) vs. 12.9	~41 (both)	NR	Poor mobilization did not compromise engraftment, PFS, or OS
Lemieux et al., 2021 (CIBMTR) [9]	Registry analysis	975	Second ASCT after upfront ASCT	9.8 (overall); 17.3 if ≥24 mo	30.9 (overall); 71.3 if ≥24 mo	2% (100 d); 4% (1 y)	Duration of remission after ASCT1 strongest predictor of benefit
Bicskó et al., 2024 [10]	Single-center retrospective	30	Late-relapse RRMM	24	48	3%	Day-100 CR/VGPR predicted superior PFS (32 vs. 8.5 months)
Čemažar et al., 2025 [2]	Single-center retrospective	78	Salvage ASCT	24 (95% CI 20–36)	76	NR	High ORR (85%); maintenance did not significantly affect outcomes
André et al., 2024 (France) [11]	Multicenter real-life	267	ASCT2 at relapse	EFS 31 mo	97 mo (8.1 y)	1%	VGPR+ and maintenance were strongest predictors of OS
Yarlagadda et al., 2021 [12]	Single-center retrospective	69	Dara-refractory RRMM	7.3	19.3	NR	Salvage ASCT achieved ORR 80%; ≥VGPR associated with longer OS
Tremblay et al., 2017 (Letter) [13]	Retrospective	74	Cytopenic, refractory RRMM	6.1	19.3	4% (100 d)	ASCT2 enabled hematologic recovery and trial eligibility despite modest PFS
Baertsch et al., 2025 (GMMG ReLApsE) [14]	Phase III randomized	139 (ASCT arm)	LEN/DEX ± ASCT	20.5 vs. 19.3	67.1 vs. 62.7	NR	No OS/PFS benefit for routine salvage ASCT; high crossover diluted effect
Gregersen et al., 2022 (Nordic CARFI) [15]	Phase II randomized	168	Salvage ASCT + maintenance	TTP 25.1 vs. 16.7	NR vs. 44.5	1.1%	Carfilzomib–dex maintenance significantly prolonged disease control

ASCT, autologous stem cell transplantation; ASCT1, first autologous stem cell transplantation; ASCT2, second autologous stem cell transplantation; auto-HCT, autologous hematopoietic cell transplantation; RRMM, relapsed or refractory multiple myeloma; PFS, progression-free survival; OS, overall survival; EFS, event-free survival; TTP, time to progression; NRM, non-relapse mortality; TRM, transplant-related mortality; ORR, overall response rate; CR, complete response; VGPR, very good partial response; PR, partial response; MRD, minimal residual disease; R-ISS, Revised International Staging System; DoR, duration of response; PM, poor mobilizer; LEN, lenalidomide; DEX, dexamethasone; CT, chemotherapy; CI, confidence interval; EBMT, European Society for Blood and Marrow Transplantation; CIBMTR, Center for International Blood and Marrow Transplant Research.

## Data Availability

No new data were created or analyzed in this study.
